# Toward a hybrid assessment framework for adolescent borderline personality disorder: a mini review of personality functioning, digital biomarkers, and AI-supported assessment

**DOI:** 10.3389/fpsyt.2026.1758535

**Published:** 2026-07-08

**Authors:** Sara Močnik, Hojka Gregorič Kumperščak

**Affiliations:** 1 Unit for Paediatric and Adolescent Psychiatry, Division of Paediatrics, University Medical Centre, Maribor, Slovenia; 2 Laboratory for Digital Signal Processing, Faculty of Electrical Engineering and Computer Science, University of Maribor, Maribor, Slovenia; 3 Department of Psychiatry, Faculty of Medicine, University of Maribor, Maribor, Slovenia

**Keywords:** artificial intelligence, borderline personality disorder, digital biomarkers, hybrid assessment, personality functioning

## Abstract

Borderline personality disorder (BPD) in adolescence is increasingly recognized as a valid and clinically meaningful diagnosis, yet it remains frequently underdetected and undertreated. Diagnostic uncertainty often arises from overlap between typical developmental features, such as emotional reactivity, identity exploration, and fluctuating peer relationships, and the pathological instability characteristic of BPD. Stigma and clinician hesitancy to diagnose before age 18 further delay intervention, despite evidence that early identification improves outcomes. Dimensional diagnostic frameworks, including the DSM-5 Alternative Model for Personality Disorders and ICD-11, define personality pathology through impairments in levels of personality functioning (LPF) across self and interpersonal domains. Validated adolescent instruments such as the LoPF-Q 12–18 and AIDA operationalize these constructs, but rely on static and retrospective self-report, limiting their ability to capture rapid, context-dependent shifts in adolescent BPD. This mini review synthesizes diagnostic challenges, evaluates LPF-based assessment tools, and highlights the need for approaches that better capture dynamic variability. Digital phenotyping and artificial intelligence (AI) offers promising adjuncts to traditional assessments. Candidate digital biomarkers from smartphones and wearables, including speech patterns, activity data, and physiological signals, may provide continuous and ecologically valid indicators relevant to personality functioning. Rather than presenting a validated diagnostic framework, this mini review proposes a conceptual direction for future hybrid assessment integrating LPF-based tools, structured clinical interviews, ecological momentary assessment, and candidate digital biomarkers.

## 1 Introduction

Borderline personality disorder (BPD) is a severe psychiatric condition characterized by pervasive instability in affect regulation, identity, and relationships, often accompanied by impulsivity (1). Adult lifetime prevalence ranges from 0.7% to 2.7% and rises to 22% in inpatient psychiatric settings (2). In adolescents, community prevalence is estimated at 1-3%, with clinical rates reaching 33-49% in inpatient and 11-22% in outpatient populations (3). Longitudinal studies show that many adolescents who meet BPD criteria continue to experience symptoms or impairment in adulthood, and even subthreshold presentations are associated with functional difficulties (4, 5).

Although early intervention improves long term outcomes, diagnostic delays of five to seven years remain common due to clinical hesitancy, stigma, and difficulties differentiating pathology from normative adolescent development (6, 7). These barriers highlight the need for more developmentally sensitive and objective assessment approaches.

The DSM 5 Alternative Model for Personality Disorders (AMPD) and ICD 11 represent a paradigm shift by defining personality pathology as impairments in levels of personality functioning (LPF) across self and interpersonal domains (8, 9). LPF deficits often precede categorical BPD, and early difficulties in self-control, impulsivity, and affective instability have been shown to predict later disorder onset (10). These vulnerabilities may trigger developmental cascades that perpetuate emotional dysregulation over time (11).

Validated dimensional measures such as the LoPF-Q 12–18 and AIDA reliably assess LPF in adolescents and are undergoing cross cultural validation (12, 13). However, these tools, like structured interviews, provide static and resource intensive evaluations that fail to capture the rapid fluctuations central to adolescent BPD (14).

While our recent scoping review (15) mapped observable cues in BPD detectable by AI, the present mini review specifically addresses early diagnostic challenges in adolescence. It integrates established LPF-based tools (LOPF-Q 12–18 and AIDA) with emerging digital approaches and outlines a conceptual hybrid pathway for clinical practice. This provides a developmentally sensitive, clinically oriented synthesis that existing reviews do not offer.

Digital phenotyping, the continuous measurement of behavioral and physiological signals from smartphones and wearables, offers a promising complement to traditional tools (16, 17). A possible pathway toward hybrid assessment would integrate LPF based instruments and clinical interviews with digitally derived data analyzed using AI to provide a more dynamic, context sensitive approach (18, 19). LPF tools remain essential as severity anchors but capture only brief snapshots of functioning, while retrospective self-report and infrequent clinical contact are vulnerable to recall bias and miss moment to moment variability (20, 21).

In line with the scope of a mini review, this article provides a focused synthesis of recent developments relevant to adolescent BPD assessment. It does not aim to present a validated clinical framework or original data. It extends previous work on observable cues in BPD by shifting the focus from cue identification to clinical integration (15). Rather than providing another systematic evidence map, it examines how candidate digital markers may be conceptually linked to adolescent personality functioning and established LPF-based assessment. Its added value lies in bringing together adolescent BPD assessment, dimensional personality functioning models, and emerging digital phenotyping/AI-supported approaches as a possible pathway toward future hybrid assessment (8, 9, 13, 17, 18, 22, 23).

## 2 Diagnostic challenges in adolescent BPD

Adolescence is marked by identity exploration, emotional lability, and shifting social relationships, processes that are normative but can also resemble features of BPD (24). Transient difficulties in identity coherence and heightened mood variability often overlap with early signs of pathology (5). While identity crisis may reflect normal development, more pervasive identity diffusion may signal emerging BPD (25). The dynamic and context dependent expression of these phenomena complicates clear differentiation between typical adolescent transitions and psychopathology instability (24).

Persistent stigma and diagnostic reluctance remain major barriers to early recognition. The misconception that BPD cannot be diagnosed before age 18 remains widespread, despite robust evidence of its validity, reliability, and clinical utility in adolescents (3, 6, 7). Clinicians often hesitate to assign the diagnosis, fearing the label may stigmatize young people or prove unstable over time (7, 26). Such hesitancy delays timely intervention, exacerbating longterm impairment (6, 26).

Comorbidity is the rule rather than the exception in adolescent BPD. In clinical samples, depressive disorders co-occur in 55–71%, ADHD in 25–60%, and PTSD in 15–25% (27, 28). These high rates increase diagnostic complexity and blur boundaries with other conditions (29–31). Most validation studies have been conducted in Western populations, limiting global generalizability. Studies from non-Western settings, including China and India, suggest that cultural factors shape both symptom expression and diagnostic interpretation (32, 33).

Gender influences both presentation and detection of BPD. Males are often underdiagnosed, potentially due to lower screening rates, differences in help-seeking behavior, or historical diagnostic bias (34, 35). Females more frequently exhibit internalizing symptoms such as affective instability and chronic emptiness, while males more commonly show externalizing behaviors including impulsivity and anger (34, 36, 37).

Together, these challenges illustrate the limitations of static categorical frameworks and support the transition to dimensional, process-oriented approaches capable of tracking dynamic changes over time.

## 3 Personality functioning models and validated assessment

The DSM-5 AMPD and ICD-11 reconceptualize personality pathology through a dimensional lens, describing disorder severity along a continuum of impairments in self and interpersonal functioning (38, 39). This model captures the core of personality pathology: impairments in identity, self-direction, empathy, and intimacy and provides a more developmentally sensitive framework for adolescent assessment (7, 40). It enables differentiation between normative developmental challenges and emerging personality dysfunction (41, 42).

### 3.1 Established instruments

The Levels of Personality Functioning Questionnaire 12–18 (LoPF-Q 12–18) measures personality disorder severity across the four LPF domains and demonstrates strong reliability, validity, and cross-cultural applicability (13, 22, 43–45).

The Assessment of Identity Development in Adolescence (AIDA) focuses on identity diffusion, a hallmark of borderline personality organization, and shows excellent psychometric properties (12).

Structured interviews such as the Childhood Interview for Borderline Personality Disorder (CI-BPD) remain diagnostic reference standards, showing good reliability though limited by time demands and developmental variability (46, 47).

While these instruments provide valuable clinical anchors, they remain limited to static, retrospective assessment, underscoring the need for more dynamic and ecologically valid methods (21, 48).

## 4 The research gap: from static to dynamic assessment

BPD in adolescence involves rapid, context-dependent shifts in emotion, behavior, and relationships (49, 50). Traditional static tools cannot capture this moment-to-moment variability (21, 51). This limitation is particularly evident in assessing emotion dysregulation, a defining feature of BPD that unfolds across moments rather than months (50, 52). Without fine-grained monitoring, clinicians may overlook early signs of deterioration or contextual triggers that could inform timely intervention.

Ecological momentary assessment (EMA) provides a bridge between static evaluation and real-world monitoring. By collecting repeated self-reports of emotions and experiences in natural contexts, EMA improves temporal resolution and reduces recall bias (21, 51, 53). EMA thus lays the groundwork for digital phenotyping, enabling continuous assessment of personality functioning and symptom variability in daily life.

## 5 Emerging directions: digital biomarkers and artificial intelligence

### 5.1 Digital biomarkers

Digital phenotyping refers to the moment-by-moment quantification of individual-level behavioral, cognitive, social, and physiological patterns using data from personal digital devices such as smartphones, wearables, and sensors (54, 55). Digital phenotyping aims to bridge the gap between episodic clinical assessment and real-world functioning by collecting objective and repeated data outside traditional clinical settings (56, 57). In psychiatry, digital biomarkers can be understood as measurable, technology-derived indicators of clinically relevant behavioral or physiological processes, including symptoms, functioning, risk states, or change over time (58–60). These data may be actively collected, for example through ecological momentary assessment, voice tasks, or self-report prompts, or passively collected through device-use patterns and sensors, including mobility, sleep, activity, communication rhythms, speech characteristics, and physiological arousal (48, 56, 61, 62).

In adolescent BPD assessment, candidate digital biomarkers may be particularly relevant because they can capture variability, context dependence, and temporal instability more directly than retrospective questionnaires (21, 48, 53). Potential strengths include ecological validity, repeated measurement, reduced recall bias, detection of within-person change, monitoring of context-dependent fluctuations, and the possibility of identifying early warning signals before clinically evident deterioration (48, 53, 61, 62). These measures may therefore support a more fine-grained assessment of dynamic processes that are difficult to capture during episodic clinical contact (21, 53, 60).

Observable cues are clinically or behaviorally observable signs that may be detected by clinicians, informants, or computational systems. In BPD, these may include language use, speech and prosodic features, facial expressions, nonverbal behavior, movement, activity patterns, interpersonal behavior, and physiological arousal (63–65). Observable cues become candidate digital biomarkers only when they are captured through digital tools, reliably quantified, and shown to have clinically meaningful associations with symptoms, functioning, risk, or treatment-relevant change (58, 60, 65). Our previous scoping review identified several observable cues in BPD, including speech and language features, facial expression, nonverbal behavior, activity patterns, and physiological arousal, that may be measurable through digital tools and could inform future AI-supported assessment (65).

Candidate digital biomarkers may be conceptually linked to LPF domains, although these links remain hypothetical and require empirical validation (15). Aspects of identity and self-functioning should not be treated as directly measurable digital signals. Rather, they may be indirectly reflected in speech and linguistic features, such as narrative coherence, narrative stability, agency, autobiographical continuity, emotional tone, and self-referential language (65–68). Self-direction may be explored through goal-directed behavior, daily routines, academic or social functioning, and smartphone activity rhythms (69, 70). Similarly, interpersonal functioning, including empathy and intimacy, may be reflected in communication rhythms, social interaction patterns, facial expression, and interactional behavior (65, 71–73). Finally, physiological indices such as heart rate variability, skin conductance, sleep, and activity patterns may provide additional markers of arousal and emotion regulation (65, 74, 75).

However, digital biomarkers should not be interpreted as diagnostically specific indicators of BPD, because similar behavioral or physiological patterns may occur in depression, anxiety, ADHD, PTSD, autism spectrum disorder, substance use, or normative adolescent stress (50, 51, 76–78). Further limitations include missing data, device non-adherence, measurement noise, privacy and consent concerns, unequal access to technology, cultural variability in communication patterns, algorithmic bias, and the risk of overinterpreting correlational signals as disorder-specific markers (60, 79–81). Digital biomarkers should therefore be conceptualized as candidate adjuncts to clinical and LPF-based assessment rather than as stand-alone diagnostic instruments (58–60, 82).

### 5.2 Artificial intelligence

AI in psychiatry refers to computational methods that learn from clinical, behavioral, biological, or digital data to support tasks such as classification, risk prediction, symptom monitoring, prognosis, and treatment planning (17, 23, 83, 84). In the context of adolescent BPD assessment, AI-supported analysis should not be viewed as a tool for automated diagnosis, but as a potential adjunct for integrating multimodal information, detecting patterns, monitoring trajectories, stratifying risk, identifying subgroups, and generating clinically testable hypotheses (17, 83–85). This distinction is important because youth mental health AI remains insufficiently validated for routine clinical use, and adolescent populations require particular attention to fairness, developmental appropriateness, clinical interpretability, and safety (83, 84, 86).

Relevant AI/machine learning(ML) approaches include supervised models, such as logistic regression, random forests, support vector machines, gradient boosting, and neural networks, for classification or risk prediction; unsupervised approaches, such as clustering or latent profile methods, for identifying clinically meaningful subgroups; and longitudinal models, such as mixed-effects models, recurrent neural networks, temporal convolutional networks, or transformer-based architectures, for modeling within-person change over time (23, 83, 84, 87). In a possible hybrid assessment pathway for adolescent BPD, supervised models could be used to estimate risk or symptom severity, unsupervised models could explore heterogeneity within BPD presentations, and longitudinal models could examine dynamic changes in affective instability, interpersonal functioning, activity, speech, or physiological arousal over time.

Methodological rigor is essential before such approaches can be translated into clinical practice (83, 84, 87, 88). Evaluation should include transparent reporting of data sources, eligibility criteria, feature extraction, missing-data handling, preprocessing, model development, internal validation, and external or prospective validation in independent adolescent samples (84, 88). Depending on the clinical task, relevant performance metrics may include sensitivity, specificity, area under the receiver operating characteristic curve, precision, recall, F1-score, calibration, and clinical utility, because discrimination alone is insufficient for clinical decision-making (87, 89–91). For adolescent psychiatry, interpretability, calibration, fairness, and clinical usability are as important as predictive accuracy, because model outputs must support rather than replace clinician-led formulation and shared decision-making (83, 84, 86, 92).

### 5.3 Ethical and clinical implications

Implementing digital and AI-based tools in youth psychiatry demands strict safeguards for data privacy, algorithmic transparency, and informed consent (79, 80, 86, 93). Bias mitigation, equity, and developmental sensitivity are essential to prevent misuse and ensure inclusivity (81, 83). Ethical frameworks such as ACCEPT-AI outline standards for consent, data protection, and communication in pediatric AI research (94). Responsible adoption must preserve the clinician–patient relationship while enhancing diagnostic insight and personalization.

## 6 A conceptual pathway toward hybrid assessment

Building on the preceding sections, this chapter translates the proposed hybrid assessment approach into a conceptual pathway for adolescent BPD assessment.

**Figure 1:**
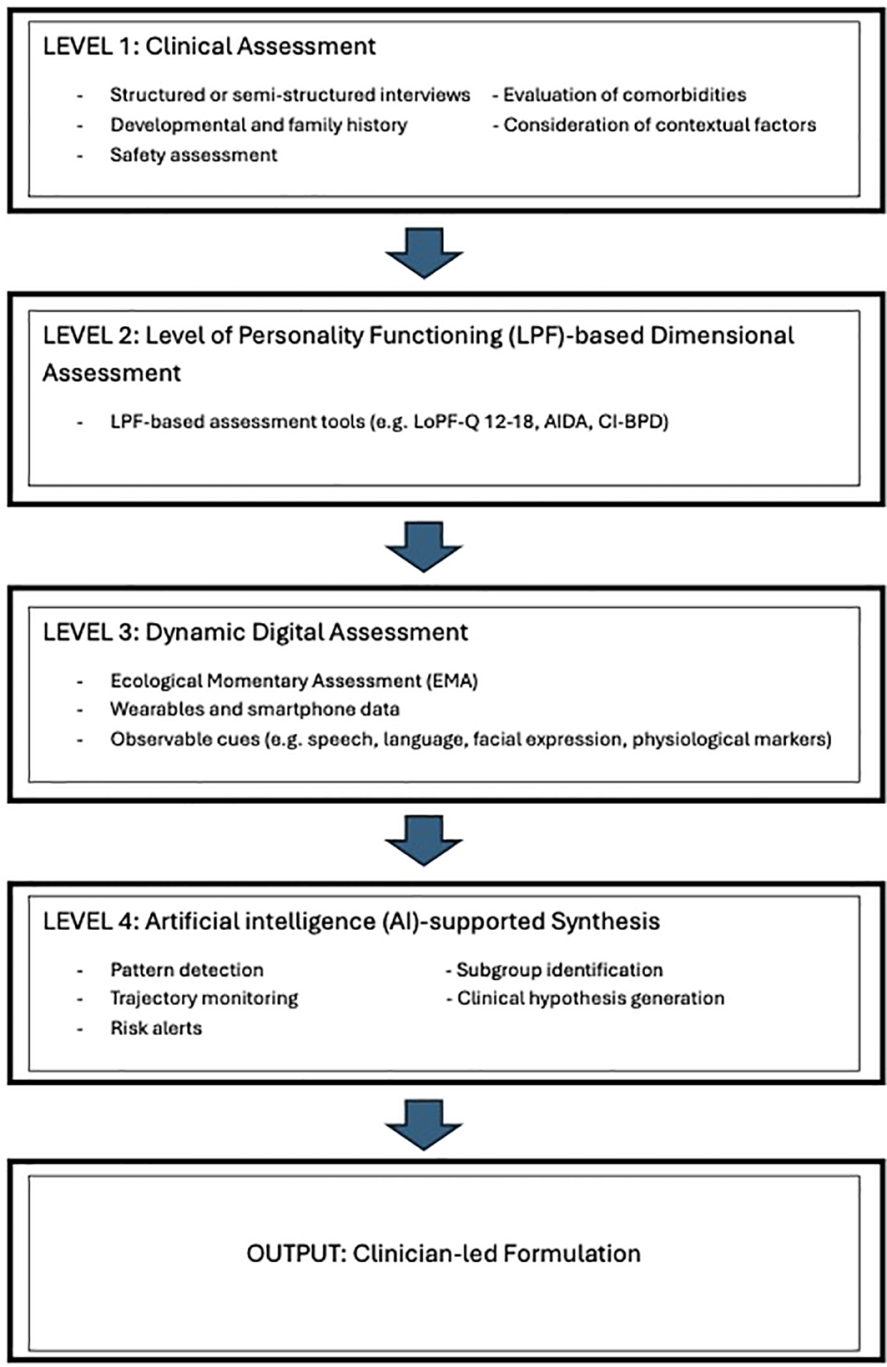
Conceptual pathway toward hybrid assessment of adolescent borderline personality disorder. The pathway integrates clinical assessment, LPF-based dimensional assessment, dynamic digital phenotyping, and AI-supported synthesis, with the final output remaining a clinician-led formulation.

As illustrated in **Figure 1**, the proposed hybrid pathway integrates four hierarchical levels.

The first level is established clinical assessment, including structured and semi-structured interviews, developmental and family history, comorbidity evaluation, and safety assessment (7, 24, 26).

The second level involves LPF-based dimensional assessment using the LoPF-Q 12–18, AIDA, and CI-BPD, which provide severity anchors for identity, self-direction, empathy, and intimacy (12, 13, 47).

The third level comprises dynamic digital phenotyping via EMA, smartphone-based behavioral data, wearables, and observable cues to capture real time variability (15, 48, 53).

The fourth level uses AI-supported synthesis for pattern detection, trajectory monitoring, risk alerts, and multimodal integration (17, 83, 84).

The output of this pathway is a clinician-led formulation that combines static LPF anchors with dynamic digital indicators. This model is proposed as a conceptual direction for future research and clinical validation rather than a ready-to-use protocol. Future studies should test feasibility, validity, acceptability, and clinical utility before implementation (80, 94).

## 7 Discussion

This mini review highlights that static categorical and LPF-based assessments alone are insufficient to capture the dynamic nature of adolescent BPD. The conceptual hybrid framework outlined above addresses the diagnostic challenges and research gaps by integrating LPF measures with digital biomarkers and AI-supported analysis.

Dimensional LPF-based models offer an important step forward because they focus on impairments in self and interpersonal functioning, which are central to adolescent personality pathology. Instruments such as the LoPF-Q 12–18 and AIDA provide developmentally relevant anchors for assessing severity and identity pathology (12, 13). However, these measures remain largely static and retrospective. They are therefore best understood as essential trait-level anchors rather than complete tools for capturing the rapid fluctuations in affect regulation, self-experience, and interpersonal functioning that characterize adolescent BPD.

EMA and candidate digital biomarkers may complement these tools by providing repeated, context-sensitive information about daily-life functioning. Smartphone-based behavioral data, speech and language features, facial-expression patterns, physiological arousal, sleep, and activity rhythms could contribute to a more dynamic understanding of individual trajectories (15, 48, 53, 61, 62). Their main potential lies not in replacing clinical assessment, but in making clinically relevant variability more observable and measurable.

AI-supported analysis may further assist by integrating multimodal information and identifying patterns across clinical, psychometric, and digital data. Such approaches may eventually support trajectory monitoring, early-warning signals, subgroup identification, and individualized intervention planning (17, 83–85). However, predictive accuracy alone is insufficient for clinical translation. Models used in adolescent psychiatry must be interpretable, calibrated, developmentally appropriate, fair across populations, and acceptable to young people, families, and clinicians.

Taken together, the proposed pathway should be viewed as a future-oriented direction for research and clinical development, intended to guide validation studies rather than immediate routine implementation. Hybrid assessment of adolescent BPD would need to preserve clinician-led formulation as its foundation, with LPF-based measures providing severity anchors and digital/AI-supported methods serving as adjunctive tools. Future studies should test feasibility, validity, acceptability, and clinical utility in diverse adolescent samples, while addressing privacy, informed assent and consent, bias, transparency, and cultural adaptation from the outset (79–81, 86, 94).

A hybrid approach to adolescent BPD assessment is promising only if it remains clinically grounded, developmentally sensitive, and ethically robust. The most realistic near-term goal is not automated diagnosis, but better integration of established clinical assessment, LPF-based instruments, and carefully validated dynamic indicators. Such integration may help clinicians move from static snapshots toward a more precise understanding of individual developmental trajectories, while preserving the central role of clinical judgment and therapeutic relationship.
